# Sciatic Integrity Is Necessary for Fast and Efficient Scrapie Infection After Footpad Injection

**DOI:** 10.3390/ijms26157273

**Published:** 2025-07-28

**Authors:** Franco Cardone, Flavia Porreca, Marco Sbriccoli, Anna Poleggi, Anna Ladogana, Mei Lu, Maurizio Pocchiari, Luigi Di Giamberardino

**Affiliations:** 1 Istituto Superiore di Sanità, Viale Regina Elena 299, 00161 Rome, Italy; marco.sbriccoli@iss.it (M.S.); anna.poleggi@iss.it (A.P.); anna.ladogana@iss.it (A.L.); meilu@sickkids.ca (M.L.); maurizio.pocchiari@iss.it (M.P.); ldg373@gmail.com (L.D.G.); 2 Department of Paediatrics, The Hospital for Sick Children (SickKids), University of Toronto, Toronto, ON M5G 1X8, Canada

**Keywords:** scrapie, prion, neuroinvasion, 263K, neurotropic pathogens, intraneural transit

## Abstract

The agents of prion diseases have the capacity to efficiently infect susceptible hosts by peripheral routes and to project to clinical target areas of the central nervous system (CNS) via peripheral nerves. Understanding the process of prion spread from the site of infection to the CNS may allow us to identify novel therapeutic strategies. To investigate the mechanism involved in the intranerval transit of 263K scrapie prions in golden Syrian hamsters (GSHs), we transected the sciatic nerve at increasing times post-footpad injection and recorded the incubation periods as estimates of the efficiency of infection. We calculated that intranerval transit of this strain of scrapie is at least 10 times faster than previously reported and may reach 50 mm/day, similar to other neurotropic viruses. By in vivo exposure/injection of sciatic nerves to 263K infectivity, we have also shown that prion entry likely occurs via nerve terminals rather than by direct contact with the sciatic nerve. Application of this experimental approach in other forms of prion diseases could allow verification of the timing of neuroinvasion, a relevant parameter for the definition of therapeutic interventions.

## 1. Introduction

The naturally occurring scrapie disease of sheep and goats is the prototype of a wider group of infectious, spontaneous, or hereditary pathologies of mammalians called transmissible spongiform encephalopathies (TSEs), or prion diseases (PDs, from the name of the proteinaceous infectious agent) [1,2].

TSEs belong to the large group of neurodegenerative disorders with amyloidosis, a relevant collection of dementing illnesses that includes Alzheimer’s disease, Parkinson’s disease, Lewy body dementia, and many others [3]. In this group, TSEs stand alone as being fully transmissible both within and between species, both in field and in experimental conditions, and by parenteral and non-parenteral routes. These properties, together with the absence of preventive/treatment methods, the absence of preclinical diagnostic alterations during the long incubation period, and the strong resistance of prions to disinfection/sterilization methods, separately or synergically operated on several occasions during the second part of the last century to put TSEs in the spotlight as a public health issue.

Even though earlier recordings of TSEs (scrapie of sheep) date back to the eighteenth century, common awareness over these deadly disorders remained beneath the level of concern until the late 1980s, when the threatening, worldwide epidemic of bovine spongiform encephalopathy (BSE) began. The BSE crisis dismantled the traditional view of animal TSEs as relatively inoffensive diseases for humans, leading to a profound review of livestock production systems and to an unprecedented acceleration of scientific efforts in the search for causes, remedies, and diagnostic tools in the whole field of prion diseases [4,5].

Results from this struggle, combined with the enforcement of specific preventive and control measures, led to a reduction in the prevalence of the epidemics in animals (within the unusually long timeframe allowed by a disease belonging to a group once associated with pathogens categorized as “slow viruses”) [6]. As immediately feared after the recognition of BSE as a new transmissible disease, consequences of the BSE outbreak had already silently crossed veterinary medicine and livestock production borders, leading, a decade later, to the rise, in the mid-1990s, of the BSE-related, zoonotic variant of Creutzfeldt–Jakob disease (vCJD) affecting people in their twenties and thirties (notably, the age at onset for sporadic CJD peaks during the sixth decade of life) [7]. A new alarm for both public health authorities and the scientific community rang out, and again, the implementation of restrictive prophylactic and preventive measures was successful, driving the decrease in the number of vCJD cases [8].

More recently, we are witnessing a novel chapter of the story, with the attention of health authorities and scientists captured by the presence of atypical forms of animal TSEs and by the diffusion, in captive and wild cervid populations in the North America and north Europe regions, of a once endemic prion disease of deer and elk called Chronic Wasting Disease (CWD) [9].

Diffusion of these forms of prion diseases mainly occurs by horizontal transmission either through direct exposure to infected animals, or following contact with contaminated objects or environment. When transmission occurs by these routes, TSE agents reach the CNS through peripheral nerves at an estimated speed (about 0.5–5 mm per day [10,11,12,13,14,15]) slower than that reported for other neurotropic agents [16,17,18,19,20,21]. The intranerval transmission rate was calculated for TSEs by measuring the time needed to detect infectivity by bioassay, or the disease marker PrP^Sc^ (the pathological isoform of the physiological prion protein PrP^C^) by immunological methods, spreading from the site of injection into the corresponding ganglia, spinal cord, or brain areas [10,11,13,14,15,22], or by measuring the difference between incubation periods of intrasciatic- and footpad (f.p.)-infected animals [12]. The slow rate of transport and the detection of periaxonal rather than intra-axonal deposits of PrP^Sc^ in peripheral nerves led to the hypothesis that prion propagation may occur along Schwann cells [12,22,23,24,25,26] or, alternatively, through a cascade of one-by-one PrP^C^ to PrP^Sc^ conversion events [3]. A similar slow rate was also observed for anterograde and retrograde spread of TSE agents in the spinal cord [11,27].

Most of these approaches, however, likely underestimate the actual speed of prion spread as they may include in their calculations at least one of the following time intervals:

(a) The time to penetrate nerve endings; (b) the time to pass through neuroanatomical structures (e.g., ganglia, spinal cord) towards the CNS; (c) the time to replicate and reach detectable levels.

The comprehension of this issue is not purely academic as the peripheral stage of TSE infection possibly represents one of the best opportunities for intervention to slow down, or even halt, the progression of prions to the CNS after peripheral infection (typical of scrapie and CWD).

To obtain further insight into this aspect, we chose to study a particular model of a hamster-adapted, experimental scrapie strain named 263K [28]. This is a specially relevant system for such studies for a number of reasons as it has a short incubation period (i.e., about 60 days after intracerebral infection with brain homogenate from diseased golden Syrian hamsters) compared to some other scrapie models, it shows a robust and reliable relationship between the infectious dose and the incubation period, and, most interestingly, it is characterized by a strong neurotropism that facilitates direct entry of the infectious agent into local nerve terminals when non-limiting infectious doses are administered [29,30].

After inoculation of golden Syrian hamsters (GSHs) with high doses of the 263K strain of scrapie into the rear footpad, infectious prions quickly penetrate into nerve endings and move intraneuronally towards the CNS [25]. As a way of measuring the rate of intranerval transit of 263K scrapie, in this study, we surgically interrupted sciatic nerve transport at increasing times post-f.p. injection and recorded the incubation periods as estimates of the efficiency of infection (the ratio of “short” and “long” incubation periods). Considering the time interval and the physical distance between the site of injection and the site of the cut, the occurrence of animals with a short incubation period would allow us to estimate the maximal theoretical speed of intranerval transit in each experimental group and to hypothesize relevant molecular motors that may then become potential pharmacological targets [31].

## 2. Results

Footpad-injected positive controls (no cut) developed scrapie with an incubation period (81.4 ± 3.0 days, mean ± SD, range 76–88, *n* = 22) similar to that previously reported with the very same experimental model [25], and negative controls (sciatic nerve cut soon after footpad injection) in 183.9 ± 25.0 days, entirely in line with already published results [32], confirming that the integrity of the sciatic nerve is crucial for the efficiency of f.p. infection with the 263K strain of scrapie (Table 1) and that 263K scrapie infectivity has the ability to follow alternative routes to the nervous system when the most efficient, or proximal, site for access is impaired.

**Figure 1:**
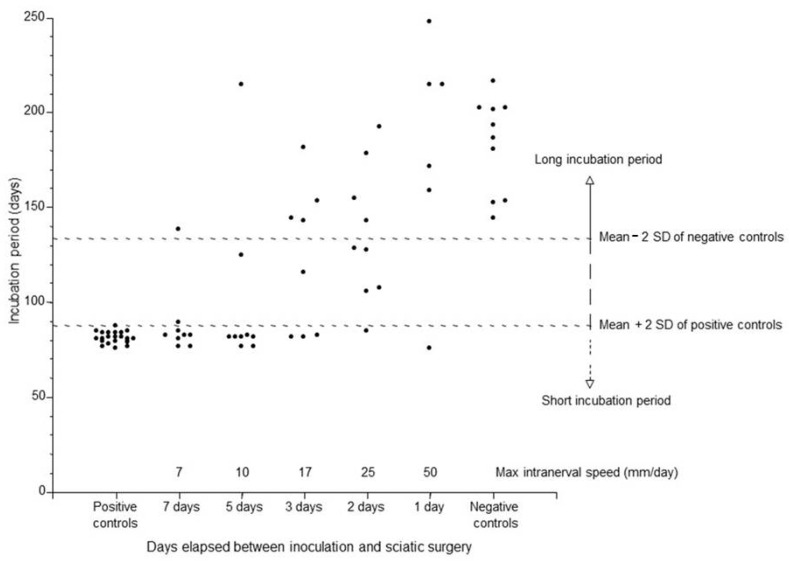
Incubation periods of Syrian hamsters after transection of the sciatic nerve at different time points after footpad infection. Each dot represents a single animal. Positive and negative controls are defined in the text.

The incubation periods of animals in the groups with the sciatic nerve cut at different time points were categorized as short (within 2SD of positive controls: 87.4 days), long (within 2SD of negative controls: 113.9 days), or intermediate (Figure 1 and Table 1). Short incubation periods indicate that the scrapie agent has efficiently reached the CNS directly via the sciatic nerve, while intermediate incubation periods likely suggest that it has reached the CNS via the sciatic nerve but with low efficiency. Long incubation periods indicate that the agent reached the CNS by a mechanism of transmission possibly different from that of the “short” group but similar to that occurring for other peripheral routes such as the intramuscular, peritoneal, or, more often, intradermal and subcutaneous routes [33].

The large majority of animals in the 7- or 5-day cut groups developed disease with a mean incubation period of about 80 days, implicating an intranerval transit rate between 7 and 10 mm/day (considering a distance of 50 mm between the site of inoculation and the sciatic cut) (Table 1). Encouraged by this outcome, we decided to test whether the speed was higher than 10 mm/day. To this aim we enlarged the number of study groups by including three groups where the sciatic nerve was cut 3 days, 2 days, and 1 day after f.p. inoculation. Interestingly, in the 3- and 2-day cut groups, about half of the animals developed scrapie with short/intermediate incubation periods, indicating that the transit speed was at least 25 mm/day. In these animals, even in the 1-day cut group, a single animal developed disease with a short incubation period. Considering that the distance between the transection point and the inoculation site is 50 mm, it is possible that a small fraction of the scrapie agent moved faster than 50 mm/day.

Since we cannot exclude that after footpad injection some parts of the inoculum diffuse upward with lymphatic flow and penetrate into nerve terminals from a site closer to the sciatic cut (thus resulting in an actual speed of retrograde transport slower than that proposed above), we set up a study to investigate how the disease develops when the site of exposure to infectious scrapie is more proximal. To this aim, we subjected the sciatic nerve located in the popliteal cavity (circa 30 mm from the footpad and 2 from the site of the sciatic cut along the femur) to either direct intranerval injection, or nerve soaking with a drop of infectious scrapie brain suspension.

As shown in Table 2, all intrasciatic-injected animals (*n* = 6) developed the disease with a mean incubation period of 86.5 days (SD = 0.5 days), whereas, in the sciatic-soaked group, only 4/6 hamsters developed clinical signs, with a mean i.p of 176.5 days (SD = 37.9; range 140–220 days). The sciatic cut produced a drop in the efficiency of infection in both groups, with only 2/12 affected hamsters in the intrasciatic group and 3/12 in the sciatic-soaked animals.

The average incubation period observed after intrasciatic injection shows a statistically significant delay of 5 days as compared with f.p.-infected, uncut animals (Student’s *t*-test, *p* < 0.0000005). It is conceivable that such an increase in the intrasciatic group results from the more precise method of administration in the intrasciatic group versus the footpad counterpart, yet is limited by the smaller volume of inoculum (1 vs. 20 μL). Both routes resulted in minimal-spread incubation periods, confirming comparable efficiencies of infection and suggesting the negligible contribution of alternative, highly efficient ports for neuroinvasion than footpad nerve terminals after f.p. injection.

## 3. Discussion

Our data confirm that after f.p. injection, the integrity of the sciatic nerve is essential for the scrapie agent to reach efficiently to the CNS [14] yet with a rate of spread higher than those previously reported in other TSE models and similar to other neurotropic viruses [16,17,18,19,20].

We observed that the probability of developing the disease with a short incubation period after footpad infection decreased with the time of sciatic transection (Figure 1), showing that the longer the interval between inoculation and cut, the higher the proportion of diseased animals presenting a short incubation period. These results may be interpreted as a demonstration that the transport of the scrapie agent into the sciatic nerve may occur in small packages of subinfectious units that need to accumulate in the corresponding spinal ganglia or spinal cord to achieve a full effective dose that induces the disease with the shortest i.p. Our hypothesis is in line with data from Chen and colleagues [34], who showed that after peripheral administration, infectious prions reach target organs in subinfectious quantities that are not able to start a successful infection. Moreover, these authors reported that this phenomenon occurs in a heterogenous manner within the same group of animals, suggesting that individual variations may result from dissimilar numbers of prions reaching the brain, from distinct rates of prion replication, or from different rates of clearance between animals or a combination of this and other factors [34]. Transection at 7, 5, 3, and 2 days produced an intermediate incubation period in some animals, suggesting that, within the timeframe allowed, the transport capacity of the sciatic nerve is not always sufficiently high to carry enough of the scrapie agent to cause disease with short incubation periods after f.p. injection. Alternatively, the rate of uptake at nerve terminals of a highly infectious 263K dose may be, sometimes, unpredictably slow. Our data cannot discriminate between these two possibilities, but they show that more than 7 days are necessary to achieve complete efficiency of infection, as can be inferred by the observation of a hamster with a “long” incubation period. In this setting, a different pathway of peripheral spread and neuroinvasion occurred, possibly recalling other infection routes where infectivity clearance/dilution may occur, with consequences for the efficiency of infection and the incubation period (e.g., intraperitoneal) [33].

The restricted CNS targeting obtainable after f.p. injection with highly neurotropic pathogens is also described in an elegant study by Marshall and colleagues, showing that in mouse, in spite of a smaller body (and footpad) size, f.p. injection of 25 µL of a herpes virus preparation precisely targets L4 and L5 dorsal root ganglia but not L3, indicating no leakage from the footpad [35].

The results we obtained with the nerve-soaked infection group showed a critically low efficiency of infection (only two thirds of exposed animals developed scrapie) and exceedingly long incubation periods that are not in contrast with the abovementioned hypothesis of a minor, if any, role played by alternative sites of entry for the scrapie agent above footpad nerve terminals. It is of note that selective intrasciatic access of infectious prions also occurs in this group, as shown by the lowered efficiency of infection when the nerve is cut shortly after exposure to prions.

The exact mechanisms of TSE agents’ entry and diffusion/propagation in the nervous system are still matters of investigation [36]. Results from different authors indicate that infectious prions gain access into nerve endings by macropinocytosis [37,38] with an efficiency independent from the size of the particles, which, conversely, affects the efficiency of intraneural propagation, with small prion aggregates more readily transported to the perikaryon than large fibrils. This latter observation is in line with the association of PrP^Sc^ oligomers with the highest specific titer of infectivity [39] and may be, at least in part, responsible for the different capacity for neuroinvasion of each prion strain.

Once they have entered, prions may be transported to cell bodies through retrograde transport [11,27], but, since impairment of these molecular motors obtained in specific experimental models [26,40] only minimally, if ever, affects neuroinvasion, it is possible that other mechanisms [32,36], yet to be identified, act either in concert or alone in prion propagation. Alternatively, it is conceivable that prion strains may exploit different, yet sometimes synergistic, ways of propagation that may prevail according to different parameters (e.g., infectious strain, state of prion aggregation, type of endocytosis, dose, route, inflammatory state, genotype) [41].

Results from our experimental setting are not in contrast with the possibility depicted by the literature of a primary mechanism of entry and dissemination into the nervous system of 263K prions comprising a first step of internalization mediated by macropinocitosys, followed by fast retrograde transport, not dissimilar to that recently proposed for reoviruses by Aravamudhan and colleagues [42].

## 4. Materials and Methods

In the f.p.-injected groups, female GSHs (*Mesocricetus auratus*, Charles River, Calco, Italy) were anesthetized with an intraperitoneal injection of xylazine (20 mg/kg, Elanco Italia, Sesto Fiorentino, Italy) and ketamine (138 mg/kg, Intervet Productions Srl, Aprilia, Italy) and injected in the footpad of the left hind leg with 20 µL of a 10% suspension in PBS of whole brains from female hamsters that were clinically ill with 263K scrapie. To produce the inoculum, whole brains were added to nine volumes of sterile PBS (pH 7.4), homogenated on ice by a motorized Teflon-glass Potter-Elvehjem tissue grinder and immediately subjected to low speed clarification at 500× *g* for 10′. At 1, 2, 3, 5, or 7 days post-infection, animals were anesthetized as above, the left sciatic nerve was exposed, and a 3 mm segment was removed at 50 mm above the footpad. Nerve and muscles were repositioned and the skin was closed with sterile surgical staples. Positive and negative control groups contained hamsters inoculated in the footpad of the left hind leg with 20 µL of the same 263K 10% brain suspension reported above and with the sciatic nerve either exposed but not cut (positive), or exposed and cut as described before (negative) 5 min after injection.

In the poplite-infected groups, animals (female) were anesthetized as above and the sciatic nerve in the popliteal space was exposed and either injected [43] or soaked with 1 µL of a 10% brain suspension from 263K scrapie-affected hamsters. When required, the sciatic nerve was cut 5 min after infection as reported above.

Animals were monitored for clinical signs of scrapie by trained personnel and humanely sacrificed soon after a full clinical diagnosis, consisting of a consistent and coherent combination of hyper-reactivity to stimuli (acoustic and tactile), hyper-reflexia, aggressiveness, wobbling head, and unsteady gait, was reached [44].

The research protocol, approved by the Centre for animal research and welfare of the Istituto Superiore di Sanità and authorized by the Italian Ministry of Health, adhered to the guidelines contained in the Italian Legislative Decree 116/92, which transposed the European Directive 86/609/EEC on Laboratory Animal Protection. The whole study was conducted under the strict supervision of veterinarians from the Centre for animal research and welfare, which ensures adherence to national and international legislation regarding animal welfare.

## 5. Conclusions

Our work supports the conjecture that prion entry in the nervous system likely occurs via nerve terminals rather than by direct contact with the sciatic nerve, and that the rate of prion spread along peripheral nerves could be faster than previously reported. It would be interesting to investigate other neurotropic TSE strains with the approach here proposed as the timing of neuroinvasion stands as one of the major determinants of the design of future preventive treatments, which are effective only before the TSE agents enter the CNS [45,46,47].

## Figures and Tables

**Table 1:** Mean incubation times of f.p.-injected animals with short, intermediate, and long incubation periods as defined in Figure 1.

	Animals with Short Incubation Periods (Days)			Animals with Intermediate Incubation Periods (Days)			Animals with Long Incubation Periods (Days)		
Cut Time	*n*	Mean ± SD	Range	*n*	Mean ± SD	Range	*n*	Mean ± SD	Range
CTRL − (5′)	0	-	-	0	-	-	10	183.9 ± 25.0	145–217
1 day	1	76	-	0	-	-	5	201.0 ± 36.1	159–248
2 day	1	85	-	4	117.8 ± 12.4	106–129	4	167.5 ± 22.6	143–193
3 day	3	82.3 ± 0.6	82–83	1	116	-	4	156.0 ± 18.0	143–182
5 day	7	80.7 ± 2.6	77–83	1	125	-	1	214	-
7 day	7	81.3 ± 3.1	77–85	1	90	-	1	139	-
CTRL + (no cut)	21	81.1 ± 2.7	76–85	1	88	-	0	-	-

**Table 2:** Incubation periods (days) of hamsters exposed to 263K infectivity at the level of the popliteal cavity.

Route	Sciatic Cut	Cut Time	Affected/Inoculated	Incubation Periods (Mean ± SD)
Intrasciatic, popliteal cavity	no	-	6/6	86.5 ± 0.5
	yes	5 min	2/12	159.5 ± 103.9
Drop, popliteal cavity	no	-	4/6	176.5 ± 37.9
	yes	5 min	3/12	219.3 ± 6.0

## Data Availability

Data are reposited in the Information processing and storage facility of the Istituto Superiore di Sanità and can be made available upon request.
